# Early Postoperative Results after Thymectomy for Thymic Cancer: A Single-Institution Experience

**DOI:** 10.1007/s00268-023-06996-5

**Published:** 2023-04-20

**Authors:** Matteo Petroncini, Piergiorgio Solli, Jury Brandolini, Giulia Lai, Filippo Antonacci, Elena Garelli, Kenji Kawamukai, Sergio Nicola Forti Parri, Barbara Bonfanti, Giampiero Dolci, Pietro Bertoglio

**Affiliations:** 1 Division of Thoracic Surgery, IRCCS Azienda Ospedaliero Universitaria Di Bologna, Via Albertoni 15, 40138 Bologna, Italy; 2 Alma Mater Studiorum, Università di Bologna, Bologna, Italy

## Abstract

**Background:**

Surgery for thymic cancers is considered the key of curative treatment. Preoperative patients’ characteristics and intraoperative features might influence postoperative outcome. We aim to verify short-term outcomes and possible risk factors for complications after thymectomy.

**Methods:**

We retrospectively investigated patients undergoing surgery for thymoma or thymic carcinoma in the period between January 1, 2008, and December 31, 2021, in our department. Preoperative features, surgical technique (open, bilateral VATS, RATS), intraoperative characteristics and incidence of postoperative complications (PC) were analyzed.

**Results:**

We included in the study 138 patients. Open surgery was performed in 76 patients (55.1%), in 36 VATS (26.1%) and in 26 RATS (36.1%). Resection of one or more adjacent organs due to neoplastic infiltration was required in 25 patients. PC appeared in 25 patients (52% Clavien–Dindo grade I, 12% grade IVa). Open surgery had a higher incidence of PC (*p* < 0.001), longer postoperative in-hospital stay (*p* = 0.045) and larger neoplasm (*p* = 0.006). PC were significant related to pulmonary resection (*p* = 0.006), phrenic nerve resection (*p* = 0.029), resection of more than one organ (*p* = 0.009) and open surgery (*p* = 0.001), but only extended surgery of more organs was confirmed as independent prognostic factor for PC (*p* = 0.0013). Patients with preoperative myasthenia symptoms have a trend toward stage IVa complications (*p* = 0.065). No differences were observed between outcomes of VATS and RATS.

**Conclusions:**

Extended resections are related to a higher incidence of PC, while VATS and RATS guarantee a lower incidence of PC and shorter postoperative stay even in patients that require extended resections. Symptomatic myasthenia patients might have a higher risk toward more severe complications.

## Introduction

Thymomas and thymic carcinomas are rare neoplasm arising from thymic epithelial cells, and although rare, they are the most frequent neoplasm of the anterior mediastinum [1, 2]. Their behavior is strictly dependent on histological features and a local infiltration of adjacent structures and organs is possible [3, 4].

Surgery is a curative treatment for thymic cancer and can be offered even in case of loco-regional metastatic diseases [2]. Recently, the technological innovation brought radical changes in the paradigms of thymic surgery: From the classical open surgery (via sternotomy, thoracotomy or clamshell incision), there was a shift toward a less invasive approach using video-assisted thoracic surgery (VATS) or robot-assisted thoracic surgery (RATS) [5, 6]. Although the superiority of the minimally invasive surgery (MIS) approach is still a matter of debate, some recent papers including two RCTs have highlighted potential advantages in terms of short-term outcomes for lung cancer patients treated with VATS compared to thoracotomy [7, 8]. The same applies to thymus surgery, where MIS has gradually become a well-established alternative approach in clinical practice, for both malignant and benign diseases, even though this approach has not been supported yet by prospective studies [2]. Nevertheless, locally aggressive disease might require extended resections of one or more surrounding organs (e.g., lung, chest wall, pericardium, phrenic nerve) increasing the difficulty of procedures that might require to be performed by open technique according to the extent of the disease and of the experience of the surgeon [4, 9].

The present study is focused on short-term surgical outcomes and associated risk factors, regardless of the technique. Data have been retrospectively collected from a single-center large institutional database across a 14-year period.

## Patients and methods

This study was approved by the ethical committee of Bologna (protocol number 721/2022/Oss/AOUBo).

### Patients

We retrospectively collected all consecutive patients who underwent surgery for thymic neoplasm (both thymoma and thymic carcinoma) at our institution from January 1, 2008, to December 31, 2021.

All patients with incomplete perioperative information were excluded.

All cases were restaged according to the eighth edition of TNM classification.

PC were recorded according to the Clavien–Dindo score [10]; when a patient experienced more than one complication of different grade, he was assigned with the highest grade.

Thirty- and 90-day mortality and hospital readmission in the first 90 days after surgery were also recorded.

### Surgical techniques

Surgical technique was decided according to the availability of MIS technologies and according to surgeon preference.

Surgery encompassed resection of the whole thymus en-bloc with surrounding fat tissue between phrenic nerves.

Open approaches included sternotomy, antero-lateral thoracotomy, cervicotomy or sternal splits, and they were performed in a standard manner. An open approach was mostly used in the first period 2008–2014 or even recently in case of large lesions with associated infiltration of surrounding structures deemed not suitable for a radical treatment with MIS surgery.

VATS technique was represented by either a unilateral or bilateral approach. Unilateral approach consisted in a standard three-portal technique with incision made around the breast; the side of the intervention was left to surgeons’ choice. Our standard bilateral approach was already described elsewhere [11].

Robotic procedures were performed using the Da Vinci^©^ platform, Xi version. Thymectomy was carried out according to standard techniques already described by other authors [5]. Although the side of intervention was based on surgeon’s preference according to anatomic features of the tumor, a left-side approach was generally preferred.

### Statistical analysis

Data were analyzed using the software SPSS version 26.0 for IOS (Chicago, USA). Continuous variables were expressed in terms of mean with standard deviation (SD) or median with range, while categorical variables were expressed in terms of frequency. Two-tailed Pearson’s Chi-square test was used for intergroup comparison of categorical variables, while the Student’s *T* test and ANOVA test were used for continuous variables. Multivariable analysis was performed with logistic binary regression only with variables which had at least a *p* value ≤ 0.05 at the univariate analysis. Variables considered for univariate analysis were those clinically relevant and that better define postoperative outcomes: age, sex, tumor dimension, extended resection, type of extended resection, preoperative myasthenia and preoperative presence of symptoms due to myasthenia. The hazard ratio (HR) and 95% confidence intervals (CI) were reported for covariates.

## Results

In the study period, 138 patients underwent thymectomy for thymoma or thymic carcinoma in our institution. Table 1 reports the main features of the population. Briefly, the majority of patients were female (73, 52.9%) and the mean age was 59.7 years (range 19–86). Respiratory comorbidities (mainly COPD) were present in 19 (13.8%) patients, while 32 had cardiovascular comorbidities; myasthenia was present in 40 patients (29.0%).

**Table 1:** Preoperative, intraoperative and postoperative features of patients of the entire cohort and comparison between those treated with open and minimally invasive surgery

	Whole cohort	Open	Minimally invasive	*p*
*Sex (n, %)*				
Male	65 (47.1)	30 (39.5)	35 (56.5)	0.035
Age (mean, range)	59.7 (19–86)	60.7 (25–86)	58.5 (19–85)	0.402
*Respiratory comorbidities (n, %)*				
Yes	19 (13.8)	12 (15.8)	7 (11.3)	0.347
*Cardiac comorbidities (n, %)*				
Yes	32 (23.2)	15 (19.7)	17 (27.4)	0.152
*Previous cancers (n, %)*				
Yes	31 (22.5)	17 (22.4)	14 (22.6)	0.506
*Pre-surgical myasthenia (n, %)*				
Yes	40 (29.0)	21 (27.6)	19 (30.6)	0.348
*Symptoms myastenia (n, %)*				
Yes	22 (15.9)	15 (19.7)	7 (11.3)	0.118
*ASA (n, %)*				
1 2 3 4	30 (21.7) 61 (44.2) 28 (20.3) 19 (13.8)	14 (18.3) 35 (46.1) 16 (21.1) 11 (14.5)	16 (25.8) 26 (41.9) 12 (19.4) 8 (12.9)	0.263
SUVmax (mean, ± SD)	8.5 (11.6)	4.9 (1.2)	13.2 (± 17.1)	0.106
Mean tumor diameter (mm, ± SD)	49.3 (± 30.0)	55.5 (± 34.2)	41.2 (± 19.6)	0.008
*TNM stage (n, %)*				
I II IIIA/B IV	109 (79.0) 6 (4.3) 8 (5.8) 15 (10.9)	51 (67.1) 6 (7.9) 6 (7.9) 13 (17.1)	58 (93.6) 0 (0.0) 2 (3.2) 2 (3.2)	0.003
*Masaoka stage (n, %)*				
I IIA/B III IV	40 (29.0) 84 (60.9) 9 (6.5) 5 (3.6)	21 (27.6) 44 (57.9) 7 (9.2) 4 (5.3)	19 (30.6) 40 (64.5) 2 (3.2) 1 (1.6)	0.471
*Histology (WHO classification)*				
A AB B1/2/3 Carcinoma	32 (23.2) 42 (30.4) 55 (39.9) 9 (6.5)	14 (18.4) 21 (27.6) 36 (47.4) 5 (6.6)	18 (29.0) 21 (33.9) 19 (30.6) 4 (6.5)	0.213
*Surgical access (n, %)*				
Open VATS RATS	76 (55.1) 36 (26.1) 26 (18.8)	NA	NA	NA
*Extended resections (n, %)*				
Yes	25 (18.1)	22 (28.9)	3 (4.8)	< 0.001
Surgical time in minutes (mean, ± SD)	139 (± 58)	137.2 (± 53.5)	141.4 (± 62.7)	0.674
Postoperative hospital stay in days (mean, ± SD)	6.3 (± 5.1)	7.1 (± 5.8)	5.2 (± 3.7)	0.045
*Postoperative complications (n, %)*				
Yes	25 (18.1)	21 (27.6)	4 (6.5)	0.001
*Clavien–Dindo complications (n, %)*				
I II III IVa	13 (52.0) 5 (20.0) 4 (16.0) 3 (12.0)	12 (15.8) 3 (3.9) 4 (5.3) 2 (2.6)	1 (1.6) 2 (3.2) 0 (0.0) 1 (1.6)	0.425

*SUVmax* maximal standard uptake value; *VATS* video-assisted thoracic surgery; *RATS* robot-assisted thoracic surgery

Open technique was used in 76 patients (55.1%), while the remaining were treated with MIS techniques: 36 VATS (15 bilateral approach, 14 right approach and 7 left approach) and 26 RATS. The mean operative time was 139.1 min (± 57.7). No significant difference was seen between patients treated with open or MIS (*p* = 0.674). Conversely, the diameter of thymomas treated with VATS or RATS was significantly smaller compared to those treated with open surgery (*p* = 0.006). Thymic carcinoma was present in 6 patients, while among thymomas B type was the most common according to WHO classification. As reported in Table 1, most of patients were in stage I according to eighth TNM staging system and to Masaoka–Koga classification. There was no difference between open and VATS regarding WHO classification (*p* = 0.213) and Masaoka–Koga (*p* = 0.471), while we found a different distribution in TNM staging (*p* = 0.003).

In 25 patients, a resection of adjacent organs was required for loco-regional infiltration: 5 patients required lung resection, 3 pericardial resection, 3 phrenic nerve resection, 1 vascular resection and 12 resection of more than one structure or organ. Among patients who underwent a phrenic nerve resection, at preoperative chest X-ray, 2 had a clear preoperative phrenic palsy, while in the remaining cases no preoperative palsy was detectable.

Extended resections were significantly more frequent in open procedures compared to those carried either by VATS or RATS (*p* < 0.001) and in patients with higher TNM stage (*p* < 0.001) and Masaoka–Koga (*p* < 0.001).

Estimation of intraoperative blood loss and evaluation of postoperative pain were outside the aims of the present study; moreover, due the retrospective setting of the study, we found a high rate of missing values that did not allow us to consider these data. All patients affected by myasthenia were admitted in the ICU for the first postoperative night for monitoring; moreover, 4 patients with cardiac comorbidities were admitted in the ICU after surgery for the same reason. All patients were discharged on the next days to the ordinary ward.

The mean postoperative stay was 6.3 days (± 5.1), and we noticed a significant shorter course for patients treated with MIS (*p* = 0.045).

Interestingly, no case of 30- or 90-day mortality was recorded. Only one patient was readmitted on postoperative day 36 due to medical reason (intestinal sub-occlusion and urinary tract infection), and none of the cases was readmitted for surgical post-discharge complications.

### Postoperative complications

During the postoperative course, 25 patients experienced PC (18.1%) and, among them, 2 (1.4%) cases needed a redo surgery. Table 2 reports the details of each complication and the corresponding Clavien-Dindo classification. PC were more common in patients operated with an open technique (*p* = 0.001), but no difference was recorded in the rate of redo surgery (*p* = 0.164). The majority of complications (13, 52.0%) had a Clavien–Dindo grade I, while grade IVa was observed in 3 cases (12.0%). In 9 patients, more than one complication was reported.

**Table 2:** Description of all postoperative complications occurred and their classification according to Clavien–Dindo classification

Complication	Clavien–Dindo classification	Number of patients
Pneumonia	I	2
Dyspnea	I	2
Pneumothorax not requiring chest drain	I	1
Hypokalemia and hypocalcemia	I	1
Fever > 38 °C	I	2
Hypertension/tachycardia	I	4
Vocal cord palsy	I	3
Hemi-diaphragmatic palsy not requiring surgery	I	1
Pleural effusion	I	3
Left arm edema	I	1
Pericarditis	II	2
Chylothorax	II	1
Anemia requiring blood transfusions	II	4
Myasthenic crisis (not requiring intubation)	II	2
Pneumothorax requiring chest drain	IIIa	1
Atrial fibrillation	IIIa	2
Hemothorax	IIIb	2
Respiratory failure requiring intubation	IVa	3

With regard to the incidence of PC, it was significantly higher in patients who underwent an extended surgery (*p* < 0.001): In particular, we notice a higher rate in those who underwent lung resection (*p* = 0.006), phrenic nerve resection (*p* = 0.029) or resection of more than one organ (*p* = 0.009); on the other hand, there was no significant correlation with pericardial or vascular resection (*p* = 0.459 and *p* = 0.816, respectively). Moreover, no correlation between surgical time and PC was noticed, even if there was trend toward a longer surgical duration (*p* = 0.069). Concurrently, preoperative features such as myasthenia (*p* = 0.256), myasthenia symptoms (*p* = 0.310), the presence of respiratory comorbidities (*p* = 0.526), cardiac comorbidities (*p* = 0.538) or both cardiac and respiratory comorbidities (*p* = 0.653) were not significantly related to PC, and neither were postoperative features such as TNM stage (*p* = 0.200), Masaoka–Koga stage (*p* = 0.192) and WHO histological classification (*p* = 0.176). On the other hand, complications were related to a significant longer postoperative length of stay (*p* < 0.001); interestingly, in this subgroup we did not observe differences in the length of postoperative course between those treated with open and MIS (*p* = 0.426).

Multivariable analysis (Table 3) confirmed that only extended resection to multiple organs (*p* = 0.013) was associated with a higher incidence of PC, even though open surgery (*p* = 0.057) and phrenic nerve resection (*p* = 0.076) were closed to significance threshold.

**Table 3:** Multivariable analysis of risk factors related to postoperative complications

Variable	HR	CI 95%	*p*
Open surgery	3.215	0.967–10.687	0.057
Pulmonary resection	7.568	0.001–15.093	0.999
Phrenic nerve resection	9.508	0.787–114.831	0.076
Multiorgan resection	**5.199**	**1.409–19.190**	**0.013**

*HR* hazard ratio; *CI* confidence interval

Bold values are statistically significant

When we looked at different grades of complications according to the Clavien–Dindo classification, we could not find any significant correlation with preoperative features of patients such as respiratory (*p* = 0.585) or cardiologic (*p* = 0.976) comorbidities, previous cancers (*p* = 0.055) or the presence of myasthenia (*p* = 0.659). Patients with preoperative symptomatic myasthenia and those who had an extended resection of multiple organs showed a trend toward a higher incidence of Clavien–Dindo IVa complications (*p* = 0.060 and *p* = 0.065, respectively) even though non-significant. Lastly, length of postoperative course reflected the grade of complication (*p* = 0.010).

### VATS/RATS comparison

We analyzed outcomes of 62 patients treated with MIS. Twenty-five patients (40.3%) were treated by RATS and 37 (59.7%) by VATS. No case of conversion to open surgery occurred in the study period. In this subgroup of patients, PC rate was 6.5% (4 patients) and they were not related to the extended resection (*p* = 0.816). Comparing VATS and RATS, we did not find any significant differences in terms of tumor diameter (*p* = 0.172), incidence of complications (0.533), grade of PC (*p* = 0.135) and rate of extended resections (*p* = 0.646). Surgical time (*p* = 0.515) and postoperative course (*p* = 0.336) were not significantly different between VATS and RATS.

## Comment

Surgery for thymoma and thymic carcinoma has remained the main treatment throughout the decades. Sternotomy and open techniques have been gradually replaced by MIS which is nowadays commonly used, especially for early-stage and non-infiltrating thymomas [12]. Feasibility and safety of MIS have been extensively assessed by multiple retrospective reports and meta-analysis [5, 13–18].

Our results confirm some of the expected benefits of the MIS compared to open techniques (regardless of sternotomy or thoracotomy or combined approach): reduced morbidity and reduced length of stay.

Conversely, the duration of surgery was similar, regardless of the chosen technique and in line with reports from the literature. In fact, length of surgery is inconsistently reported throughout the literature and MIS does not always guarantee faster surgical operations. Marulli and colleagues [5] comparing transsternal and robotic thymectomy found a significantly longer surgical time for patients treated with the latter technique, that become non-significant after a propensity score match; similar results were more recently reported by Hurd [19] in a cohort of patients affected by thymic carcinomas. On the other hand, Casiraghi [14] found a significant longer surgical time in open procedures compared to robotic ones.

In the case of locally infiltrating lesions, the radical resection is still feasible in many cases but requires *en-bloc* excision of surrounding organs (lung, pericardium, phrenic nerve, vessels) [3, 20, 21]. Several studies reported outcomes of extended resections, but with an overall focus on long-term outcomes, with only few concentrated on early postoperative morbidity.

In the present series, extended resections were associated with a significant higher incidence of immediate complications and were more often approached with an open technique than MIS.

Also according to the literature, the role for MIS in the case of extended surgery for thymic neoplasms is still controversial and remains mainly correlated with anatomical features, abilities of surgeon and multidisciplinary attitude [9]. Soder and co-workers [22] retrospectively analyzed a monocentric 20-year experience in thymic surgery comparing open and robotic approach: They find a significant reduction in PC in the robotic group (6.8% versus 21.1%) despite a significant greater proportion of extended resections in the robotic group after a propensity score match. Conversely, in a small retrospective study, Chen [23] compared VATS and open surgery in the treatment of Masaoka stage III thymomas and they found that phrenic nerve and vena cava resection was more likely to be performed by open surgery rather than VATS. Nonetheless, in the vast majority of reported experiences, complex extended resections are performed by open sternotomy, thoracotomy or clamshell incision [3, 9, 21, 24, 25].

Concurrently, Kang [26] reported outcome of 59 patients with stage III thymic cancers which received open extended resection; he reported 5 patients with PC and he highlighted that patients that needed resection of more than one organ had a significant worse survival compared to those who had resection of a single organ. In our study, multiple organs involvement/resection had a significant longer surgical time and in-hospital course and it was the sole independent risk factor for PC.

In our experience, the overall PC rate was 18.1%, with most of the complications recorded in the context of extended surgery. Almost half of them were minor morbidities (stage I according to Clavien–Dindo) and only 3 (12%) were major (stage IVa).

In the subgroup of MIS complication rate was significantly decreased (6.5%) consistent with the report from Soder [22]. Notably, we did not observe any significant correlation between patients’ preoperative features and the severity of the complications. Although non-significant, we recorded a mild trend of stage IVa complications in patients with preoperative symptomatic myasthenia. In keeping with these observations, Kumar [27] found a significant higher incidence of PC in myasthenia patients compared to non-myasthenic in a series of thymoma operated on via robotic technique.

Recently, Wu [28] compared results of robotic and VATS thymectomies, not identifying significant differences in terms of overall duration of the surgical procedure. O’Sullivan and co-workers [6] found overall similar outcomes between RATS and VATS, while a more recent contribute [29] highlighted shorter length of stay and reduced complication rate in the RATS subgroup.

Data of our experience also include the initial development of the robotic program and therefore the learning curve period. We can speculate that any difference in surgical times and in postoperative in-hospital stay could be also affected from our growing experience making the subgroups not homogenous and underestimating one of the variables analyzed. The role of the learning curve on operative time has been also reported also by other authors [30, 31]. Moreover, this might be entitled by the gradual introduction of an ERAS approach in our institution.

Our data do not allow to precisely and undoubtedly asses which technique offers the best postoperative results in the treatment of thymus cancers, especially for complex cases. Based on our data and our experience, we can speculate that robotic technique might have the widest potential to allow to safely perform complex extended resection with good postoperative outcomes. Nevertheless, it is of paramount importance to evaluate each case singularly based on surgeon’s personal skill and experience, patient’s anatomical features and available technologies.

Our study presents some limitations. Firstly, the retrospective setting might have impaired the data collection and their quality. Secondly, the observation covers a long-time frame (14 years), where different standards of postoperative care were applied. It is worthwhile to mention the introduction of the concept of ERAS late in the 2010’s that has been applied especially to both MIS techniques and oppositely was not routine practice in the early phases when most of the patients were receiving a standard open approach. Third, the learning curves phase of each new technique, as discussed, might potentially have introduced additional bias and influenced the results. Eventually, the number of patients of our series of case and the single-center nature of the analysis offers additional limitations.

On the other hand, to the best of our knowledge, our study is the first to report a comprehensive report from a single high-volume institutional database focusing on and comparing short-term results of different surgical approaches for thymic cancers.

## Conclusions

In conclusion, we found that thymic surgery extended to more than one surrounding organ is a risk factor for PC. We also confirmed that MIS techniques, both VATS and RATS, are related to a lower incidence of PC and shorter postoperative length of stay even in patients that require extended resections. Our data did not show any preoperative features that might foresee the grade of PC, but we observe a possible tendency for more severe complications in patients with symptomatic myasthenia. Prospective and large studies are needed to confirm our findings.
